# Television Viewing and Its Association with Sedentary Behaviors, Self-Rated Health and Academic Performance among Secondary School Students in Peru

**DOI:** 10.3390/ijerph14040383

**Published:** 2017-04-05

**Authors:** Bimala Sharma, Rosemary Cosme Chavez, Ae Suk Jeong, Eun Woo Nam

**Affiliations:** 1 Yonsei Global Health Center, Yonsei University, Wonju 26493, Korea; bimalasharma@gmail.com (B.S.); rosemaryjyj@gmail.com (R.C.C.); 2 Department of Health Administration, Graduate School, Yonsei University, Wonju 26493, Korea; 3 Department of Nursing, Cheongju University, Cheongju City 28503, Korea; pubhealth@hanmail.net

**Keywords:** television viewing, sedentary behaviors, self-rated health, self-reported academic performance

## Abstract

The study assessed television viewing >2 h a day and its association with sedentary behaviors, self-rated health, and academic performance among secondary school adolescents. A cross-sectional survey was conducted among randomly selected students in Lima in 2015. We measured self-reported responses of students using a standard questionnaire, and conducted in-depth interviews with 10 parents and 10 teachers. Chi-square test, correlation and multivariate logistic regression analysis were performed among 1234 students, and thematic analysis technique was used for qualitative information. A total of 23.1% adolescents reported watching television >2 h a day. Qualitative findings also show that adolescents spend most of their leisure time watching television, playing video games or using the Internet. Television viewing had a significant positive correlation with video game use in males and older adolescents, with Internet use in both sexes, and a negative correlation with self-rated health and academic performance in females. Multivariate logistic regression analysis shows that television viewing >2 h a day, independent of physical activity was associated with video games use >2 h a day, Internet use >2 h a day, poor/fair self-rated health and poor self-reported academic performance. Television viewing time and sex had a significant interaction effect on both video game use >2 h a day and Internet use >2 h a day. Reducing television viewing time may be an effective strategy for improving health and academic performance in adolescents.

## 1. Introduction

Many children today are growing up in an obesogenic environment that causes overweight and obesity [1]. The adolescent obesity epidemic is a global issue; it is evident that a wide range of factors can contribute to obesity among children, but of growing concern is the potential contribution made by children’s media use in the present context [2,3]. Numerous studies have established the relationship between obesity and television viewing in children and adolescents [4,5,6,7,8]. Now, it is also known that high sedentary behavior can be an independent risk factor for obesity even among the people who are sufficiently physically active [9]. Researchers have proposed various theories to explain the relationship between obesity and television viewing: Television viewing encourages excess calorie intake because people eat during television watching [10], increases consumption of energy-dense food, which includes food that are more often advertised on television, and decreases consumption of fruits and vegetables [11,12,13], decreases physical activity (PA) [14] and reduces resting energy expenditure [3].

In addition, daily television viewing in excess of 2 h is associated with reduced physical and psychosocial health in children and adolescents [15]. Studies show screen-based media use including television is associated with poorer general health, and complaints such as poor vision [16], depression and anxiety [17]. Perceived health status was negatively related to screen-based media sedentary behavior in North America and Europe in adolescents [18]. A study conducted in Lima, Peru, showed that PA was associated with self-rated health [19]. Low screen time and high PA combination had the highest odds ratio for good self-rated health [20]. Similarly, screen exposure had an indirect effect on poor school performance through increased sensation-seeking among U.S. adolescents [21]. Most of the previous studies of screen-based media use focused on viewers’ eating behaviors [10,11,12,22], obesity [4,8,23], physical inactivity [14,24], and academic performance especially among children [25]. However, few studies have been conducted to test whether television viewing is independently associated with self-rated health (SRH), academic performance, and other screen-based sedentary behaviors among adolescents.

Peru is an upper-middle income country that has experienced steady economic growth in the last decade [26]. Recent studies show that the prevalence of overweight and obesity was relatively high in children and adolescents [27,28]. As stated by Busse and Ramon in their study on television viewing and eating habits of children in Lima, there is very little evidence on television viewing and associated behaviors of adolescents [29]. In addition, adolescents are more vulnerable to sedentary behaviors and poor SRH [19,27,28,29]. This study is interested in specifically exploring if television viewing is independently associated with other screen-based sedentary behaviors, SRH and academic performance in adolescents attending school. Thus, we assessed the prevalence of television viewing >2 h a day and its association with other screen-based sedentary behaviors, SRH, and self-reported academic performance (SAP) in adolescents. The study also explored the perspective of parents and teachers on adolescent’s screen-based sedentary behaviors to supplement the findings of the questionnaire survey.

## 2. Methods

### 2.1. Study Design, Area and Sampling

A cross-sectional survey was conducted among secondary school students from six public schools in the Lima Metropolitan Area (Province of Lima and Callao), Peru. The survey was done by Yonsei Global Health Center in partnership with the Korea International Cooperation Organization (KOICA) in November 2015. The study used a part of the survey information. The study participants were selected from four districts: Comas (Province of Lima), Bellavista, Ventanilla, and Mi Peru (Province of Callao). Three schools were selected from three areas of Comas: Santa Luzmila II, Laura Rodriguez and Carlos Phillips. Three schools were taken from the districts in the Callao Province, one from each district. All secondary classes were taken from each school as strata. Students were chosen from each class using systematic random sampling with a random start. The sample size was calculated using a formula recommended by Naing et al. for a prevalence study [30]. A design effect (deff) of 3 was used to correct the sampling variance. Thus, the sample size = Z^2^ × P × (1 − P)/d^2^ × deff, where the Z = Z statistic for a level of confidence (Z = 1.96), P = expected prevalence (*p* = 0.5), d = precision (d = 0.05) and deff = design effect (deff = 3). Therefore, the calculated sample size was 1153. To minimize the effect of non-responses and missing information, an additional 18% of the calculated sample was added i.e., 1354. However, 1234 students, who provided their responses on the question of “How much time do you spend during a typical or usual day watching television?” were selected for the analysis in the study.

### 2.2. Variables Measurement

#### 2.2.1. Television Viewing, Screen-Based Sedentary Behaviors and Physical Activity (PA)

A study by Leatherdale et al., found that self-reported measures of PA and sedentary behavior had test-retest reliability and criterion validity among students and suggested that they are acceptable and appropriate for use in a large-scale school surveys [31]. We used single–item self-report questions based on the Global school-based student health survey (GSHS) for PA and sedentary behavior [32]. “During a typical or normal day, how many hours do you spend watching television?” The similar type question was asked for the time using video games and the Internet. The options were <1 h, 1 to 2 h, 3 to 4 h, 5 to 6 h, 7 to 8 h and >8 h a day; the responses were coded from 1 to 6. For logistic regression analysis, the boundaries for prevalence estimates of television viewing were set at >2 h per day as high viewing, based on guidelines from the American Academy of Pediatrics [33]. Self-reported PA was measured with the following question: “During the last 7 days, how many days did you do any type of physical activity for a total of at least 60 min?”[32]. The options provided ranged from 0 day to 7 days a week.

#### 2.2.2. Self-Rated Health (SRH) and Self-Reported Academic Performance (SAP)

SRH is a valid and reliable measure among those without cognitive impairment [34]. SRH was assessed by a single question using a Likert scale type measure [34], “In general, how would you rate your health”? The response options were: excellent, very good, good, fair and poor, and the responses were coded from 1 for poor to 5 for excellent. A previous study suggested that perceived school performance item seems to be a valid and useful question to distinguish good grade students and others [35]. For SAP, we asked the following question, as it was used in the study by Kim et al. [36]: “In the last 12 months, how do you rate your academic performance”? The options were: high, above average, average, below average and poor. The responses were coded from 1 for poor to 5 for high.

### 2.3. Data Collection

A self-administered standard questionnaire was provided to the selected students in their respective classrooms during regular school hours. Ten trained enumerators gave a brief orientation on the study objective and explained how to fill out the questionnaire. As a tool for information collection, the GSHS questionnaire was modified and translated into Spanish so that the information could be collected from the students in their own language. The GSHS questionnaire is a self-administered questionnaire to assess behavior risk factors and protective factors in adolescents [32]. This survey was voluntary and anonymous. Teachers and school staff were not allowed to communicate with the students while they were completing the questionnaire. We did not provide any incentive for the students to participate in the survey. 

### 2.4. Statistical Analysis

Statistical Package for Social Science (SPSS) version 21 (IBM Corp, Armonk, NY, USA) was used for the analysis of the data. Proportions of variables were calculated to describe the characteristics of the study population. A chi-square test was used to assess the association between television viewing >2 h a day and other variables. Spearman’s correlations was also applied to assess the correlation between television viewing, and video game use, Internet use, PA, SRH, and SAP. The level of significance was set at 5% for all analysis. Multivariate logistic regression analysis was applied to find out the association between television viewing as an independent variable, and video game use, Internet use, SRH and SAP as dependent variables adjusting with sex, age group, grade, school and PA. Adjusted odds ratios (AORs) with corresponding 95% confidence intervals (CI) were presented. The model fitness was assessed using the Hosmer and Lemeshow goodness of fit test; all models had a good fit with the selected variables, with *p*-values > 0.05. 

### 2.5. Collection and Analysis of Qualitative Information

Twenty in-depth interviews were conducted: 10 among parents and 10 among school teachers from 1 to 19 August 2016. The participants for interview were selected purposively from parents at least having one adolescent child and teachers teaching in secondary level education. Face to face interview was conducted at home with parents and at school with teachers. Parents were interviewed about leisure time activity of their children, availability of television and Internet at home, and type of programs their children watch on the television. Screen-based sedentary behaviors of students and perceived barriers of controlling them were interviewed with teachers. The interviews were carried out by one of the authors of the study in Spanish. All interviews were audio recorded. All audio recordings and original notes were translated into English before analysis. Data was analyzed using the thematic analysis technique. Each interview was carefully and repeatedly read to organize the responses into similar themes. 

### 2.6. Ethical Approval

We obtained ethical approval for the study from the Institutional Review Board of the Wonju Campus of Yonsei University (IRB Number: 1041849-201510-BM-092-03) and the DIRESA Callao (Peru). Consent was also given by each school administration and from the participants’ parents or guardians in advance. Informed assent was provided by each individual student before they filled out the questionnaire. Consent was also obtained from all participants of the in-depth interviews.

## 3. Results

### 3.1. Findings from the Questionnaire Survey

Table 1 shows the characteristics of the study population. More than half, 55.8% respondents were in the age group of 11–14 years, and 38.6% were male. Of the total, 2.6% and 28.4% perceived their health as poor and fair respectively. About half, 47.8% reported that their academic performance was average and 4.6% poor. A 32.9% of the respondents reported they did PA for of at least 1 h each day for 5 or more days in the last one week. Of the total, 23.1% watched television >2 h a day, 29.9% used Internet >2 h a day and 9.2% played video games >2 h a day.

Table 2 shows the proportion of television viewing >2 h a day and association with other variables. A significant difference was observed between television viewing, and video game use, Internet use, PA, SRH, and SAP. 

Television viewing had a significant positive correlation with video game use and Internet use, but a negative correlation with PA, SRH and SAP in the total sample (Table 3). Television viewing had a significant positive correlation with video game use in males and older adolescents, with Internet use in both sexes; and a significant negative correlation with SRH and SAP in females.

Table 4 shows a logistic regression analysis of television watching >2 h a day, television viewing and sex interaction and television viewing and age group interaction as independent variables, and video game use and Internet use as dependent variables. Here, television viewing >2 h a day was coded “1” and ≤2 h “0”; male sex was coded “1”and female “0”; and age 11–14 years “1” and 15–19 years “0” to compute interaction variables. And, PA was categorized as <5 days and ≥5 days a week for the analysis.

Television viewing >2 h, while adjusting for sex, age group, PA, school and grade, was independently associated with video games use >2 h a day and Internet use >2 h a day. Adolescents who watched television in excess of 2 h per day were 2.37 times more likely to use video game >2 h a day, and 1.68 times more likely to use the Internet >2 h a day as compared to those who watched it 2 or less than 2 h per day. Significant interaction effect of television viewing time and sex was observed for both video games use and Internet use each day. Interaction effect of television viewing time and age group was not found significant. Male respondents who watched television >2 h a day were 3.2 times more likely to report video game use >2 h a day, and 2.36 times more likely to report Internet use >2 h a day than other respondents. School and grade were entered into the model as dummy variables.

Table 5 shows logistic regression analysis of television watching >2 h a day as an independent variable and SRH and SAP as dependent variables. Excellent, very good and good health was categorized into “good”, and fair and poor health into “poor/fair” for SRH. For SAP, high, above average and average were classified into “satisfactory”, and below average and poor into “poor”. 

The odds of poor/fair SRH was 1.38 among those who watched television >2 h than those who watched ≤2 h, independent of sex, age group, PA, school and grade. Similarly, while adjusting with all variables, television viewing >2 h/day was independently associated with poor SAP (AOR, 1.72; CI, 1.23 to 2.42). Grade and school were entered into the model as dummy variables.

### 3.2. Findings from In-Depth Interviews

All families we interviewed mentioned that they had a television at their home. Most of the parents reported that their children watched television throughout the day depending on their school schedule and homework load; those studying during the morning watched mostly in the afternoons and evenings; and those studying during the afternoon watched television in the mornings and evenings as well. Most of them mentioned their children watched television ≥2 h each day; and longer time during the weekends. Most parents and teachers mentioned “Reality Shows” were most common choice of content for most students, and parents affirmed to see no advantage of such programs that their children watch. Most of the parents believe the television watching takes time away from homework and concerned that children could imitate bad behaviors and learn distasteful information. However, as reported by some teachers parents themselves leave television on while leaving for work, so that children do not leave the house. In addition, the majority of parents reported watching television together with their adolescent children after they have dinner, especially movies and dramas. 

Most families did not have access to the Internet, but children sometimes go to an Internet cabin or use mobile devices to use the Internet. Most of the parents preferred television to the Internet as they could monitor what their children were watching on television much better than on the Internet. Sometimes students told their parents that they needed to go to an Internet cabin to do school work, but they really went to play and check their social networks. Most teachers expressed beliefs that the Internet is thwarting students’ creativity and development of good study habits because students copy and paste responses to assignments, some teachers also concerned risk of pedophilia and pornography. The majority of teachers stated that parents usually do not set limits for their children regarding screen-based behaviors, and these limits are essential for controlling their behavior at home. Some parents do not want their children to go outside to play because they feel it is not safe. They also afraid their children might meet drug abusers, befriend other children that could encourage bad behavior or could get involved in a fight. Some parents mentioned that they do not have time to watch what their children are doing in their leisure time.

## 4. Discussion

About one-fourth of the students watched television >2 h each day in the study area. This finding was almost similar to a study of United States (25%) [37]. According to a study conducted in New Zealand, 37.4% of 11 to 14 year-old adolescents spent >2 h per day watching television [22]. Our result was lower than this finding. Discrepancies might be due students replacing television watching for video game and Internet use. Evidence from Brazil shows that even though the prevalence of television viewing has decreased in the last decade, there is an increase in computer/video game use [38]. Television viewing did not differ across sex or age groups in the study. Qualitative findings also show that adolescents spend most of their leisure time watching television, playing video games or using the Internet. A study among parents in Lima also showed that, on average, children watch about 5 h of television on weekdays and more during weekend-days [29]. This indicates there is a gap in the amount of television watching time as reported by adolescents themselves and by the parents.

Television viewing had a significant positive correlation with video game use and Internet use, especially in male and older adolescents. A study reports that gender differences are significant in prevalence of screen- based behavior except in the television viewing [39]. Logistic regression analysis also shows that television viewing >2 h a day was significantly associated with video games >2 h a day and Internet use >2 h a day. As well as, a significant interaction effect of television viewing time and sex was observed for both video games use and Internet use each day. The finding suggests that television watching in excess of 2 h by male adolescents was more likely to be associated with playing video games >2 h a day and using the Internet >2 h a day. Male adolescents are more vulnerable for multiple sedentary behaviors than female students.

Television viewing had a significant negative correlation with PA (even though the effect size was small) in the total sample. A review study of PA and health in young people showed that PA was unrelated to television viewing [40]. However, some other studies have shown a significant association, although with a small effect size [14,41]. 

Television viewing had a significant negative correlation with SRH in female adolescents. Previous studies found that male adolescents were less likely to report poor/fair self-rated health and health complaint as compared to female [19,39]. Adolescents conceptualize health as a construct related to medical, psychological, social, and lifestyle factors [42]; which may have an influence on gender. Screen-based media use was negatively correlated with SRH in a European study including North America [18]. In the current study, logistic regression analysis shows that adolescents who watched television in excess of 2 h were more likely to report poor/fair SRH than those who watched television ≤2 h a day. Similarly, adolescents exceeding 2 h of screen time, including all types of screen media, had 30% greater odds of sub-optimal SRH among Canadian adolescents [43].

In the current study, television viewing was negatively correlated with SAP. Logistic regression analysis shows that adolescents who watched television in excess of 2 h were more likely to report poor academic performance than those who watched television ≤2 h a day. The students who watched television for a longer duration might have spent less time doing homework, studying and reading, which may have led to decreased academic performance. A similar finding was reported in a study by Kantomaa et al. in Finland that active adolescents were about twice as likely to have high grade-point average compared with sedentary television viewers [44]. Regarding the mechanisms for the impact of visual media, a study conducted among U.S. youth explains that visual media use adversely affect school performance by increasing sensation seeking, substance use, and school problem behavior [21]. A review article also stated that watching television for >2 h per day was associated with decreased academic achievement [15]. 

Based on the study, it can be recommended that decision makers and planners at schools and related institutions need to be aware of the significance effect of watching television in excess of 2 h a day and prepare appropriate measures.

### Limitation of the Study

This study is valuable as a rarely conducted study of the relationship between television viewing and its association with other screen-based sedentary behaviors, SRH, and academic performance among secondary school adolescents. Despite these advantages, however, our study was subject to several limitations. First, as the data are cross-sectional, we could not infer causality. Second, as all of the measurements including academic performance were based on self-report, there may be methodological bias. 

## 5. Conclusions

About one-fourth of the students surveyed reported they watched television >2 h a day. Qualitative findings also show that adolescents spend most of their leisure time watching television, playing video games or using the Internet. Television viewing had a significant positive correlation with video game use in male and older adolescents, with Internet use in both sexes, and negative correlation with SRH and SAP in females, with PA in older adolescents. Multivariate logistic regression analysis shows that adolescents who watched television >2 h a day were 2.37 times more likely to use video game >2 h a day, and 1.68 times more likely to use the Internet >2 h a day than those who watched television ≤2 h. Similarly, adolescents who watched television in excess of 2 h a day were 1.38 times more likely to report poor/fair SRH, and 1.72 times more likely to report poor academic performance. Also, television viewing time and sex had a significant interaction effect on video game use >2 h a day and Internet use >2 h a day. Reducing television watching time could be an effective strategy for improving health and school performance in adolescents. 

## Figures and Tables

**Table 1:** Characteristics of the study population.

Variables	Number (*n* = 1234)	Percent (%)
Age (in years)		
11–14	689	55.8
15–19	545	44.2
Sex		
Male	476	38.6
Female	758	61.4
Self-rated health		
Poor	32	2.6
Fair	350	28.4
Good	476	38.6
Very good	239	19.4
Excellent	133	10.8
Missing	4	0.3
Self-reported academic performance		
Poor	57	4.6
Below average	139	11.3
Average	590	47.8
Above average	284	23.0
High	158	12.8
Missing	6	0.5
Days of physical activity (PA) in last week		
0 (none)	133	10.8
1	201	16.3
2	174	14.1
3	202	16.4
4	109	8.8
≥5	406	32.9
Missing	9	0.7
Television viewing per day		
Less than 1 h	564	45.7
1 to 2 h	385	31.2
3 to 4 h	194	15.7
5 to 6 h	50	4.1
7 to 8 h	16	1.3
≥8 h	25	2.0
Internet use per day		
Less than 1 h	350	28.4
1 to 2 h	398	32.3
3 to 4 h	196	15.9
5 to 6 h	81	6.6
7 to 8 h	38	3.1
≥8 h	49	4.0
Missing	122	9.9
Video game use per day		
Less than 1 h	504	40.8
1 to 2 h	179	14.5
3 to 4 h	64	5.2
5 to 6 h	30	2.4
7 to 8 h	6	0.5
≥8 h	13	1.1
Missing	438	35.5

**Table 2:** Television viewing and its association with screen-based sedentary behaviors, PA, SRH and SAP.

Variables	Television Viewing per Day (*n*, %)		
	≤2 h	>2 h	*p*-Value
Total (*n* = 1234)	949 (76.9)	285 (23.1)	
Age (in years)			
11–14	539 (78.2)	150 (21.8)	0.214
15–19	410 (75.2)	135 (24.8)	
Sex			
Male	371 (77.9)	105 (22.1)	0.493
Female	578 (76.3)	180 (23.7)	
Video game use per day			
≤2 h	540 (79.1)	143 (20.9)	0.000
>2 h	72 (63.7)	41 (36.3)	
Internet use per day			
≤2 h	598 (79.9)	150 (20.1)	0.001
>2 h	259 (71.2)	105 (28.8)	
PA (days in a week)			
0–1	237 (71.0)	97 (29.0)	0.024
2	133 (76.4)	41 (23.6)	
3	166 (82.2)	36 (17.8)	
4	88 (80.7)	21 (19.3)	
≥5	318 (78.3)	88 (21.7)	
SRH			
Excellent	99 (74.4)	34 (25.6)	0.006
Very good	195 (81.6)	44 (18.4)	
Good	376 (79.0)	100 (21.0)	
Fair	257 (73.4)	93 (26.6)	
Poor	18 (56.3)	14 (43.8)	
SAP			
High	134 (84.8)	24 (15.2)	0.000
Above average	224 (78.9)	60 (21.1)	
Average	453 (76.8)	137 (23.2)	
Below average	99 (71.2)	40 (28.8)	
Poor	33 (57.9)	24 (42.1)	

PA: physical activity, SRH: self-rated health, SAP: self-reported academic performance.

**Table 3:** Correlation of television viewing with sedentary behaviors, PA, SRH, PBI and SAP.

Variables	Total	Males	Females	11–14 Years	15–19 Years
Video game use	0.114 **	0.257 ***	−0.015	0.076	0.166 **
Internet use	0.157 ***	0.234 ***	0.105 **	0.133 **	0.176 ***
PA	−0.080 **	−0.064	−0.085 *	0.047	−0.115 **
SRH	−0.079 **	0.022	−0.133 ***	−0.064	−0.095 *
SAP	−0.111 ***	−0.068	−0.136 ***	−0.116 **	−0.102 *

PA: physical activity, SRH: self-rated health, SAP: self-reported academic performance; *** *p* < 0.001, ** *p* < 0.01, * *p* < 0.05.

**Table 4:** Adjusted odds ratios of video game use and Internet use for television viewing >2 h a day.

Variables	Video Game Use >2 h per Day ^a^ AOR (95% CI)	Internet Use > 2 h per Day ^a^ AOR (95% CI)
Television viewing >2 h per day	2.37 (1.52−3.70) ***	1.68 (1.24−2.26) **
Television viewing and sex interaction	3.20 (1.85−5.54) ***	2.36 (1.45−3.85) ***
Television viewing and age group interaction	1.57 (0.84−2.95)	1.40 (0.91−2.15)

**^a^** Adjusted for sex, age group, physical activity, school and grade; AOR: adjusted odds ratio, CI: confidence interval; *** *p* < 0.001, ** *p* < 0.01.

**Table 5:** Adjusted odds ratios of poor/fair SRH, and poor SAP for television viewing >2 h a day.

Variables	Poor/Fair SRH ^a^ AOR (95% CI)	Poor SAP ^a^ AOR (95% CI)
Television viewing		
>2 h	1.38 (1.03–1.85) *	1.72 (1.23–2.42) **
≤2 h	1	1
Sex		
Female	2.52 (1.87–3.41) ***	1.45 (1.01–2.09) *
Male	1	1
Age group		
11–14 years	0.75 (0.50–1.13)	0.56 (0.34–0.91) *
15–19 years	1	1
PA		
<5 days	1.49 (1.12–1.98) **	0.90 (0.64–1.26)
≥5 days	1	1

^a^ Variables entered were television viewing, sex, age group, PA, school and grade; PA: physical activity, SRH: self-rated health, SAP: self-reported academic performance, AOR: adjusted odds ratio, CI: confidence interval; *** *p* < 0.001, ** *p* < 0.01, * *p* < 0.05.

## References

[1] World Health Organization (WHO) Ending Childhood Obesity. http://apps.who.int/iris/bitstream/10665/204176/1/9789241510066_eng.pdf?ua=1.

[2] Janssen I., Katzmarzyk P.T., Boyce W.F., Vereecken C., Mulvihill C., Roberts C., Currie C., Pickett W. (2005). Comparison of overweight and obesity prevalence in school-aged youth from 34 countries and their relationships with physical activity and dietary patterns. Obes. Rev..

[3] Jordan A.B., Robinson T.N. (2008). Children, television viewing, and weight status: Summary and recommendations from an expert panel meeting. Ann. Am. Acad. Political Soc. Sci..

[4] Davison K.K., Marshall S.J., Birch L.L. (2006). Cross-sectional and longitudinal associations between TV viewing and girls’ body mass index, overweight status, and percentage of body fat. J. Pediatr..

[5] Fuller-Tyszkiewicz M., Skouteris H., Hardy L.L., Halse C. (2012). The associations between TV viewing, food intake, and BMI. A prospective analysis of data from the Longitudinal Study of Australian Children. Appetite.

[6] Ghavamzadeh S., Khalkhali H.R., Alizadeh M. (2013). TV viewing, independent of physical activity and obesogenic foods, increases overweight and obesity in adolescents. J. Health Popul. Nutr..

[7] Kaur H., Choi W.S., Mayo M.S., Harris K.J. (2003). Duration of television watching is associated with increased body mass index. J. Pediatr..

[8] Lowry R., Wechsler H., Galuska D.A., Fulton J.E., Kann L. (2002). Television viewing and its associations with overweight, sedentary lifestyle, and insufficient consumption of fruits and vegetables among U.S. high school students: Differences by race, ethnicity, and gender. J. Sch. Health.

[9] Sugiyama T., Healy G.N., Dunstan D.W., Salmon J., Owen N. (2008). Joint associations of multiple leisure-time sedentary behaviours and physical activity with obesity in Australian adults. Int. J. Behav. Nutr. Phys. Act..

[10] Wiecha J.L., Peterson K.E., Ludwig D.S., Kim J., Sobol A., Gortmaker S.L. (2006). When children eat what they watch: Impact of television viewing on dietary intake in youth. Arch. Pediatr. Adolesc. Med..

[11] Buijzen M., Schuurman J., Bomhof E. (2008). Associations between children’s television advertising exposure and their food consumption patterns: A household diary-Survey study. Appetite.

[12] Falbe J., Willett W.C., Rosner B., Gortmaker S.L., Sonneville K.R., Field A.E. (2014). Longitudinal relations of television, electronic games, and digital versatile discs with changes in diet in adolescents. Am. J. Clin. Nutr..

[13] Scully M., Dixon H., White V., Beckmann K. (2007). Dietary, physical activity and sedentary behaviour among Australian secondary students in 2005. Health Promot. Int..

[14] Koezuka N., Koo M., Allison K.R., Adlaf E.M., Dwyer J.J., Faulkner G., Goodman J. (2006). The relationship between sedentary activities and physical inactivity among adolescents: Results from the Canadian Community Health Survey. J. Adolesc. Health.

[15] Tremblay M.S., LeBlanc A.G., Kho M.E., Saunders T.J., Larouche R., Colley R.C., Goldfield G., Gorber S.C. (2011). Systematic review of sedentary behaviour and health indicators in school-aged children and youth. Int. J. Behav. Nutr. Phys. Act..

[16] Bener A., Al-Mahdi H.S., Vachhani P.J., Al-Nufal M., Ali A.I. (2010). Do excessive Internet use, television viewing and poor lifestyle habits affect low vision in school children?. Child. Health Care.

[17] Maras D., Flament M.F., Murray M., Buchholz A., Henderson K.A., Obeid N., Goldfield G.S. (2015). Screen time is associated with depression and anxiety in Canadian youth. Prev. Med..

[18] Iannotti R.J., Janssen I., Haug E., Kololo H., Annaheim B., Borraccino A. (2009). Interrelationships of adolescent physical activity, screen-based sedentary behaviour, and social and psychological health. Int. J. Public Health.

[19] Sharma B., Nam E.W., Kim D., Yoon Y.M., Kim Y., Kim H.Y. (2016). Role of gender, family, lifestyle and psychological factors in self-rated health among urban adolescents in Peru: A school-based cross-sectional survey. BMJ Open.

[20] Matin N., Kelishadi R., Heshmat R., Motamed-Gorji N., Djalalinia S., Motlagh M.E., Ardalan G., Arefirad T., Mohammadi R., Safiri S. (2016). Joint association of screen time and physical activity on self-rated health and life satisfaction in children and adolescents: The CASPIAN-IV study. Int. Health.

[21] Sharif I., Wills T.A., Sargent J.D. (2010). Effect of visual media use on school performance: A prospective study. J. Adolesc. Health.

[22] Utter J., Scragg R., Schaaf D. (2006). Associations between television viewing and consumption of commonly advertised foods among New Zealand children and young adolescents. Public Health Nutr..

[23] Gomez L.F., Parra D.C., Lobelo F., Samper B., Moreno J., Jacoby E., Lucumi D.I., Matsudo S., Borda C. (2007). Television viewing and its association with overweight in Colombian children: Results from the 2005 National Nutrition Survey: A cross sectional study. Int. J. Behav. Nutr. Phys. Act..

[24] Proctor M.H., Moore L.L., Gao D., Cupples L.A., Bradlee M.L., Hood M.Y., Ellison R.C. (2003). Television viewing and change in body fat from preschool to early adolescence: The Framingham Children’s Study. Int. J. Obes..

[25] Smpokos E.A., Linardakis M., Papadaki A., Lionis C., Kafatos A. (2012). Secular trends in fitness, moderate-to-vigorous physical activity, and TV-viewing among first grade school children of Crete, Greece between 1992/93 and 2006/07. J. Sci. Med. Sport.

[26] The World Bank World Bank Country and Lending Groups. https://datahelpdesk.worldbank.org/knowledgebase/articles/906519#Upper_middle_income.

[27] Preston E.C., Ariana P., Penny M.E., Frost M., Plugge E. (2015). Prevalence of childhood overweight and obesity and associated factors in Peru. Rev. Panam. Salud. Publica.

[28] Nam E.W., Sharma B., Kim H.Y., Paja D.J., Yoon Y.M., Lee S.H., Kim E.H., Oh C.H., Kim Y.S., Song C.H. (2015). Obesity and hypertension among school-going adolescents in Peru. J. Lifestyle Med..

[29] Busse P., Díaz R. (2016). What are the television viewing and eating habits of children in Peru?. Glob. Health Promot..

[30] Naing L., Winn T., Rusli B.N. (2006). Practical issues in calculating the sample size for prevalence studies. Arch. Orofac. Sci..

[31] Leatherdale S.T., Laxer R.E., Faulkner G. (2014). Reliability and Validity of the Physical Activity and Sedentary Behaviour Measures in the COMPASS Study.

[32] World Health Organization Global School-Based Student Health Survey (GSHS), Questionnaire Modules. http://www.who.int/chp/gshs/GSHS_Core_Modules_2013_English.pdf.

[33] Bar-On M.E., Broughton D.D., Buttross S., Corrigan S., Gedissman A., de Rivas M.R.G., Rich M., Shifrin D.L., Brody M., Wilcox B. (2001). Children, adolescents, and television. Pediatrics.

[34] Bombak A.E. (2013). Self-rated health and public health: A critical perspective. Front. Public Health.

[35] Felder-Puig R., Griebler R., Samdal O., King M.A., Freeman J., Duer W. (2012). Does the school performance variable used in the International Health Behavior in School-Aged Children (HBSC) Study reflect students’ school grades?. J. Sch. Health.

[36] Kim S.Y., So W.Y. (2012). The relationship between school performance and the number of physical education classes attended by Korean adolescent students. J. Sports Sci. Med..

[37] Lowry R., Lee S.M., Fulton J.E., Kann L. (2009). Healthy people 2010 objectives for physical activity, physical education, and television viewing among adolescents: National trends from the Youth Risk Behavior Surveillance System, 1999−2007. J. Phys. Act. Health.

[38] Silva K.S., da Silva Lopes A., Dumith S.C., Garcia L.M.T., Bezerra J., Nahas M.V. (2014). Changes in television viewing and computers/videogames use among high school students in southern Brazil between 2001 and 2011. Int. J. Public Health.

[39] Husárová D., Veselská Z.D., Sigmundová D., Gecková A.M. (2015). Age and gender differences in prevalence of screen based behaviour, physical activity and health complaints among slovak school-aged children. Cent. Eur. J. Public Health.

[40] Biddle S.J., Gorely T., Stensel D.J. (2004). Health-enhancing physical activity and sedentary behaviour in children and adolescents. J. Sports Sci..

[41] Marshall S.J., Biddle S.J., Gorely T., Cameron N., Murdey I. (2004). Relationships between media use, body fatness and physical activity in children and youth: A meta-analysis. Int. J. Obes..

[42] Breidablik H.J., Meland E., Lydersen S. (2008). Self-rated health in adolescence: A multifactorial composite. Scand. J. Soc. Med..

[43] Herman K.M., Hopman W.M., Sabiston C.M. (2015). Physical activity, screen time and self-rated health and mental health in Canadian adolescents. Prev. Med..

[44] Kantomaa M.T., Stamatakis E., Kankaanpaa A., Kajantie E., Taanila A., Tammelin T. (2016). Associations of physical activity and sedentary behavior with adolescent academic achievement. J. Res. Adolesc..

